# A Fatal Outcome in a Case of Necrotizing Fasciitis

**DOI:** 10.7759/cureus.79181

**Published:** 2025-02-17

**Authors:** Filipa Monteiro, Tatiana Correia, Maria João Miguel, Leticia Santos, Inês Pintassilgo

**Affiliations:** 1 Internal Medicine, Hospital Garcia de Orta, Almada, PRT; 2 Infectious Diseases, Hospital Garcia de Orta, Almada, PRT

**Keywords:** fascia, klebsiella pneumoniae, multi-disciplinary teams, multi-drug resistant bacteria, necrotizing fasciitis, septic shock

## Abstract

We report the case of a 55-year-old female with a history of invasive urothelial carcinoma, hepatic and lymph node metastases, and prior radical cystectomy. The patient was admitted with a two-day history of fever, two weeks after undergoing percutaneous nephrostomy. Upon examination, she presented with hypotension, tachycardia, and abdominal pain. She was found to have severe lactic acidosis, acute renal failure, and a necrotizing soft-tissue infection confirmed via imaging. Blood cultures identified a multi-drug resistant *Klebsiella pneumoniae* infection, and the patient was treated with fluids, sodium bicarbonate, and meropenem. Despite aggressive management, the patient developed refractory septic shock and died within hours of admission. This case underscores the rapid progression and lethality of necrotizing fasciitis, even in the absence of overt skin changes, highlighting the critical importance of early recognition and intervention in such high-risk infections.

## Introduction

Necrotizing soft tissue infection (NSTI), including necrotizing fasciitis (NF), is a severe and rapidly progressive infection of the subcutaneous tissues, fascia, and muscles, often leading to widespread tissue destruction. Although rare, NSTIs can escalate to life-threatening conditions within hours to days, particularly in immunocompromised individuals or those with significant comorbidities [1-3]. The infection typically presents with localized pain, fever, and signs of sepsis, but it can advance without overt skin changes, complicating early diagnosis and timely intervention. Recognizing predisposing factors, such as underlying malignancy or immunosuppressive treatments, is crucial in identifying high-risk patients. We present the case of a patient with a history of urothelial carcinoma, hepatic and lymph node metastases, who developed an extremely aggressive form of NF following percutaneous nephrostomy. The rapid progression of the infection and her unfavorable response to treatment underscore the importance of high clinical suspicion and early diagnosis in the management of this potentially fatal condition.

## Case presentation

We present the case of a 55-year-old female with a history of invasive urothelial carcinoma with hepatic and lymph node metastases, who had undergone radical cystectomy two years earlier.

The patient was admitted to the emergency department with a two-day history of fever, two weeks after undergoing percutaneous nephrostomy. Physical examination revealed hypotension, tachycardia, maintained urinary output through the nephrostomy, and diffuse abdominal pain with guarding.

Arterial blood gas analysis revealed severe lactic acidosis. Blood tests showed leukocytosis with a predominance of neutrophils, normal platelet values, prolonged prothrombin time, acute renal failure, elevated lactate dehydrogenase, and creatine kinase, as well as C-reactive protein (Table 1). The diagnostic workup included blood and urine cultures and an abdominal and pelvic CT scan.

**Table 1:** Blood tests results.

Blood tests	Results	Reference range
pH	6.9	7.35-7.45
HCO3-	4.5 mEq/L	22-26 mEq/L
Lactate	18.5 mmol/L	<1.0 mmol/L
Leukocyte	25.5 x 10^9/L	4.0-10.0 x 10^9/L
Neutrophils	83%	40.0-80.0%
International Normalized Ratio	2.75	1.0-1.3
Creatinine	2.5 mg/dL	0.7-1.2 mg/dL
Urea	175 mg/dL	16.0-48.0 mg/dL
Lactate dehydrogenase	6390 UI/L	100-250 UI/L
Creatine Kinase	372 UI/L	<170 UI/L
C-reactive protein	34 mg/dL	<0.20 mg/dL

**Figure 1:**
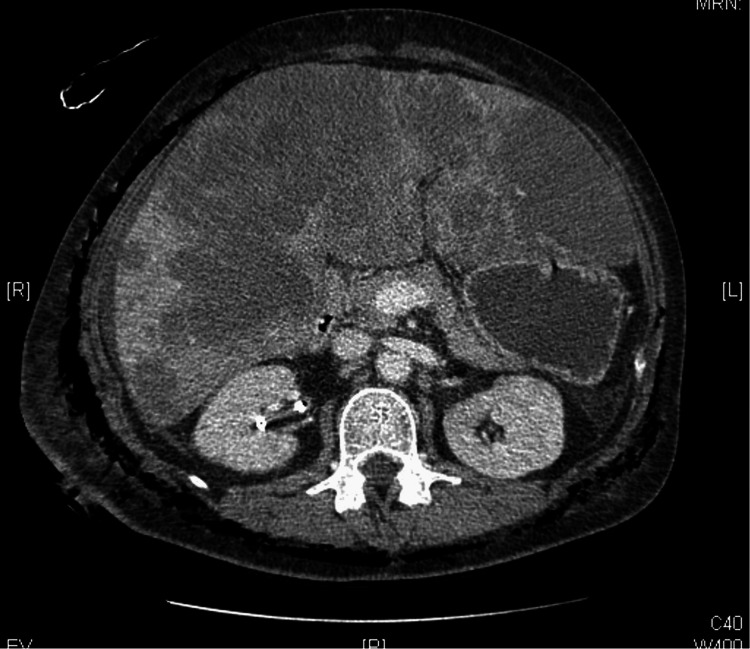
Necrotizing soft-tissue infection involving nearly the entire body circumference.

**Figure 2:**
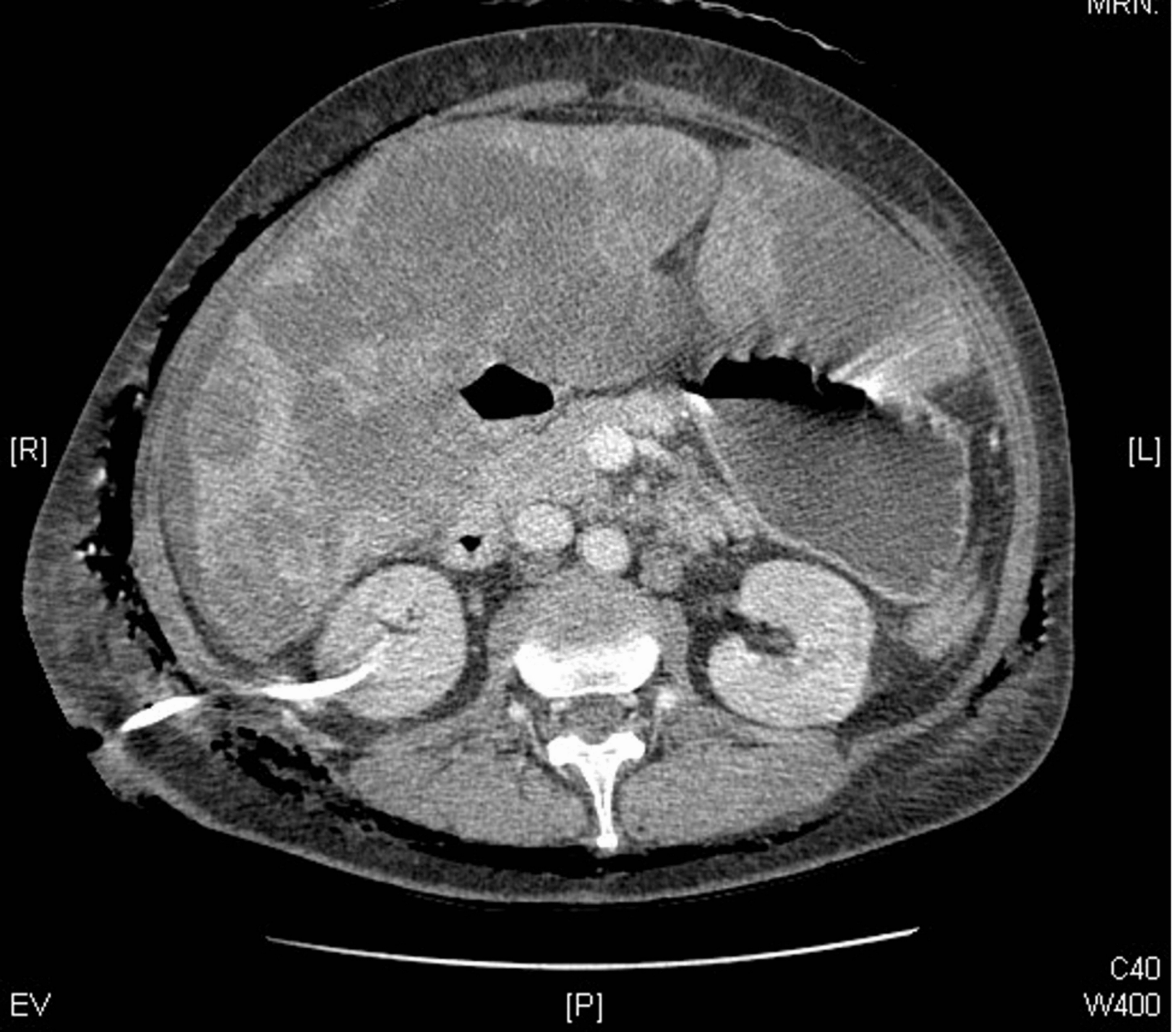
Necrotizing soft-tissue infection involving nearly the entire body circumference, with percutaneous nephrostomy trajectory.

The patient received fluids, sodium bicarbonate, and meropenem, based on a previously isolated multi-drug resistant *Klebsiella pneumoniae* in a urine culture. The CT scan revealed a necrotizing soft-tissue infection encircling the entire body (Figures 1-2).

Due to her unfavorable clinical condition and prognosis, surgical treatment was considered futile. The patient rapidly progressed to refractory septic shock and died a few hours later.

## Discussion

NSTIs, including NF, are rare but life-threatening conditions characterized by the rapid spread of infection through subcutaneous tissue, fascia, and muscle, often leading to extensive tissue necrosis and systemic sepsis [4]. While NF can be caused by a variety of microorganisms, including both aerobic and anaerobic bacteria, *Klebsiella pneumoniae* has been increasingly recognized as a causative agent, especially in patients with underlying malignancies or immunosuppressive conditions [5]. The case presented here highlights an unusually aggressive form of NF in a 55-year-old female with advanced urothelial carcinoma and metastases, who developed the infection following percutaneous nephrostomy.

In this patient, the onset of symptoms, including fever, hypotension, tachycardia, and abdominal pain, occurred shortly after the nephrostomy. The diagnosis of NF was supported by imaging findings of necrotizing soft-tissue infection affecting nearly the entire body circumference. These features, coupled with severe laboratory abnormalities such as lactic acidosis, leukocytosis, acute renal failure, and elevated lactate dehydrogenase and creatine kinase, confirmed the presence of a systemic, fulminant infection.

The rapid progression of the infection in this case is consistent with the aggressive nature of NSTIs, which can spread quickly through fascial planes and lead to significant organ dysfunction and sepsis. In many cases, skin changes may not be immediately apparent, which can delay diagnosis and appropriate intervention. Early recognition of NF and prompt initiation of treatment are critical for improving patient outcomes. In this patient, despite aggressive resuscitation and antibiotic therapy with meropenem, the infection rapidly escalated to refractory septic shock, and the patient ultimately died within hours of admission. This underscores the poor prognosis associated with extensive NSTIs, particularly in patients with advanced malignancies, where immune function is already compromised and the body’s ability to mount an effective response to infection is diminished.

The presence of malignancy, particularly urothelial carcinoma with metastases, may predispose patients to NSTIs through a combination of factors, including immunosuppression from cancer treatments, presence of indwelling devices such as urostomies, and the potential for local ischemia in areas affected by tumor burden. Furthermore, the emergence of multi-drug resistant organisms, such as *Klebsiella pneumoniae*, adds another layer of complexity to the management of these infections, requiring broad-spectrum antibiotics and, in some cases, the need for surgical debridement.

This case also highlights the importance of clinical vigilance in patients with cancer and those who have undergone recent surgery or procedures. A high level of suspicion for NSTI should be maintained in patients presenting with systemic signs of infection, especially when associated with localized symptoms such as pain, swelling, or fever. Diagnostic workup should include appropriate imaging, blood and wound cultures, and consideration of early surgical consultation for debridement if the diagnosis of NSTI is suspected. The rapid escalation of the infection in this case emphasizes the need for early and aggressive management, even in patients with poor prognoses, as delays in treatment can lead to irreversible organ damage and death.

## Conclusions

Although rare, NF represents a severe and rapidly progressive clinical entity that demands high clinical suspicion and swift intervention. This case report describes an extremely aggressive case of NF, highlighting the importance of a high level of suspicion and early diagnosis, particularly in vulnerable patients with underlying malignancy, who are predisposed to NSTIs due to factors such as immunosuppression, surgical procedures, and indwelling devices like nephrostomy/urostomy. The rapid onset and fulminant course of the infection observed in this patient, exacerbated by the presence of multi-drug resistant *Klebsiella pneumoniae*, illustrate the catastrophic consequences that can arise from delayed diagnosis and treatment in high-risk individuals. Despite aggressive management, including resuscitation and broad-spectrum antibiotics, the patient’s rapid deterioration and death highlight the poor prognosis associated with NSTIs in patients with immunosuppression. This case emphasizes the importance of early recognition, appropriate imaging, and urgent surgical consultation to prevent irreversible tissue damage and organ failure. Furthermore, it draws attention to the increasing role of resistant organisms, such as *Klebsiella pneumoniae*, in complicating the treatment of these infections. Ultimately, this report serves as a reminder of the critical need for heightened clinical vigilance in the management of immunocompromised patients, especially those with recent surgical procedures or indwelling devices. Early diagnosis and intervention remain essential to improving outcomes in these rapidly progressing, life-threatening infections.
